# Plasma Metabolites Predict Severity of Depression and Suicidal Ideation in Psychiatric Patients-A Multicenter Pilot Analysis

**DOI:** 10.1371/journal.pone.0165267

**Published:** 2016-12-16

**Authors:** Daiki Setoyama, Takahiro A. Kato, Ryota Hashimoto, Hiroshi Kunugi, Kotaro Hattori, Kohei Hayakawa, Mina Sato-Kasai, Norihiro Shimokawa, Sachie Kaneko, Sumiko Yoshida, Yu-ichi Goto, Yuka Yasuda, Hidenaga Yamamori, Masahiro Ohgidani, Noriaki Sagata, Daisuke Miura, Dongchon Kang, Shigenobu Kanba

**Affiliations:** 1 Department of Clinical Chemistry and Laboratory Medicine, Graduate School of Medical Sciences, Kyushu University, Fukuoka, Japan; 2 Department of Neuropsychiatry, Graduate School of Medical Sciences, Kyushu University, Fukuoka, Japan; 3 Innovation Center for Medical Redox Navigation, Kyushu University, Fukuoka, Japan; 4 Molecular Research Center for Children's Mental Development, United Graduate School of Child Development, Osaka University, Osaka, Japan; 5 Department of Psychiatry, Osaka University Graduate School of Medicine, Osaka, Japan; 6 Department of Mental Disorder Research, National Institute of Neuroscience, National Center of Neurology and Psychiatry, Tokyo, Japan; 7 Translational Medical Center, National Center of Neurology and Psychiatry, Tokyo, Japan; 8 National Center of Neurology and Psychiatry Hospital, Tokyo, Japan; Chiba Daigaku, JAPAN

## Abstract

Evaluating the severity of depression (SOD), especially suicidal ideation (SI), is crucial in the treatment of not only patients with mood disorders but also psychiatric patients in general. SOD has been assessed on interviews such as the Hamilton Rating Scale for Depression (HAMD)-17, and/or self-administered questionnaires such as the Patient Health Questionnaire (PHQ)-9. However, these evaluation systems have relied on a person’s subjective information, which sometimes lead to difficulties in clinical settings. To resolve this limitation, a more objective SOD evaluation system is needed. Herein, we collected clinical data including HAMD-17/PHQ-9 and blood plasma of psychiatric patients from three independent clinical centers. We performed metabolome analysis of blood plasma using liquid chromatography mass spectrometry (LC-MS), and 123 metabolites were detected. Interestingly, five plasma metabolites (3-hydroxybutyrate (3HB), betaine, citrate, creatinine, and gamma-aminobutyric acid (GABA)) are commonly associated with SOD in all three independent cohort sets regardless of the presence or absence of medication and diagnostic difference. In addition, we have shown several metabolites are independently associated with sub-symptoms of depression including SI. We successfully created a classification model to discriminate depressive patients with or without SI by machine learning technique. Finally, we produced a pilot algorithm to predict a grade of SI with citrate and kynurenine. The above metabolites may have strongly been associated with the underlying novel biological pathophysiology of SOD. We should explore the biological impact of these metabolites on depressive symptoms by utilizing a cross species study model with human and rodents. The present multicenter pilot study offers a potential utility for measuring blood metabolites as a novel objective tool for not only assessing SOD but also evaluating therapeutic efficacy in clinical practice. In addition, modification of these metabolites by diet and/or medications may be a novel therapeutic target for depression. To clarify these aspects, clinical trials measuring metabolites before/after interventions should be conducted. Larger cohort studies including non-clinical subjects are also warranted to clarify our pilot findings.

## Introduction

Depression is a state of low mood and demotivated condition that affects a person's feelings, cognition and, behaviors [1]. Major depressive disorder (MDD) and bipolar disorder (BD) are the two major depressive illnesses in psychiatry, while people with many other psychiatric illnesses can express depressive moods [2]. Severe depressive mood is recognized to be a high risk factor of suicide [3, 4], thus evaluating the severity of depression (SOD), especially suicidal ideation (SI), is crucial in psychiatric and also primary care settings [5]. The 17-item Hamilton Rating Scale for Depression (HAMD-17), rated by trained psychiatrists/psychologists, is a well-established semi-structured interview to assess the SOD [6, 7]. On the other hand, in primary care, the Patient Health Questionnaire (PHQ)-9 is utilized as one of the most reliable self-rated scales to assess the SOD [8, 9]. Present SOD evaluation systems including HAMD-17 and PHQ-9 have completely relied on a person’s subjective descriptions, spoken information and attitudes, and errors based on such subjective information are unavoidable [5]. Some persons may not express depressive mood at all in order to hide internal turmoil, and others may hyperbolically show their depressed mood to obtain a *sick role*. These difficult situations have resulted in confusion in clinical practice [10, 11]. To our knowledge, no significant blood biomarkers to predict SOD have existed until now. To resolve this limitation, objective SOD evaluation methods have been warranted.

In the field of physical illnesses, reliable and versatile blood biomarkers have been established such as HbA1c for detecting diabetes and CRP for inflammation level. These biomarkers have widely contributed to various health settings such as screening, diagnostic systems, severity ratings, and also evaluating therapeutic efficacy in clinical practice. Over the last few years, metabolomics has been utilized to develop novel biomarkers as an exploratory research tool not only for physical illness but also mental illness [12]. We have recently revealed that some metabolites are associated with MDD from a metabolomic analysis using cerebrospinal fluid (CSF) samples [13]. To our knowledge, no significant blood biomarkers to predict SOD have so far been identified with substantial multi-center validation.

The microglia hypothesis for psychiatric disorders including depression has been proposed by researchers around the world including our research team, suggesting that maladaptive microglial activation may induce various psychiatric symptoms [14–17]. From a small volume of liquid, metabolomic analysis can simultaneously measure more than 100 metabolites including several tryptophan-kynurenine pathway metabolites, which are recently known to be involved in brain inflammation and microglial activation [18, 19]. Thus, we consider metabolomic analysis to be a useful tool not only for non-target exploratory research but also for clarifying our microglia hypothesis. As the first step, we herein investigated whether some metabolites from peripheral blood of psychiatric patients are correlated with the SOD at three independent clinical centers.

## Methods

The study was approved by the ethics committee of all the three institutions (Kyushu University, Osaka University, and National Center of Neurology and Psychiatry) and was conducted in accordance with the Declaration of Helsinki. Written informed consent was obtained from all patients.

**Fig 1:**
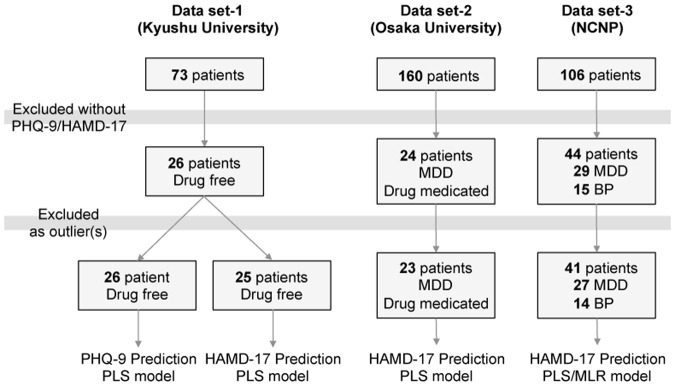
Flow diagram processing patients’ plasma samples from three independent clinical centers.

Digging up blood metabolites to predict SOD, we collected blood plasma of psychiatric patients from three independent clinical research centers; Kyushu University (26 medication-free patients with depressive mood), Osaka University (23 medicated patients who diagnosed with major depressive disorders (MDD)), and NCNP/NCNP Biobank (41 medicated/non-medicated patients who diagnosed with MDD (27 patients) and bipolar disorder (14 patients)) (Fig 1).

SOD was assessed using the Japanese version of HAMD-17 (all three centers) and PHQ-9 (only in Kyushu University) in parallel with peripheral blood collection by venipuncture. All of HAMD-17 scores were rated by trained psychiatrists/clinical psychologists. Patients with any depressive symptoms (HAMD-17 > 0) were included in this study. Aqueous metabolites in plasma were subjected to metabolome analysis using liquid chromatography mass spectrometry (LC-MS).

### Participants

#### Data set 1 (Medication-free psychiatric patients)

For the first set of samples, 26 medication-free patients for at least 2 months, who mostly were visiting a psychiatric service for the first time, were enrolled from 73 recruited psychiatric patients in Kyushu University Hospital and its related affiliations (mainly outpatients clinics) (Fig 1). Psychiatric diagnosis was made by trained psychiatrists, according to the Structured Clinical Interview for DSM-IV (SCID) [20].

#### Data set 2 (Medicated MDD)

Patients were recruited at Osaka University hospital and its related affiliations. Blood samples were collected and plasma was used for the analysis. Plasma collection of 160 psychiatric patients including MDD patients (Data set 2) was sent to Kyushu University. 24 patients with medicated-MDD who expressed depressive mood were included (Fig 1). Each patient had been diagnosed and assessed by at least two trained psychiatrists in Osaka University hospital according to SCID.

#### Data set 3 (medicated/non-medicated MDD and BD)

Patients were recruited at the NCNP Hospital, Tokyo, Japan from the NCNP Biobank. Plasma collection of 106 healthy volunteers and psychiatric patients including MDD/BP patients in NCNP (Data set 3) was sent to Kyushu University, and 29 MDD and 15 BD patients were selected for analysis (Fig 1). All participants underwent a structured interview using the Mini-International Neuropsychiatric Interview (M.I.N.I.), Japanese version [21, 22], administered by trained psychologists or psychiatrists. For participants with MDD and BD, a consensus diagnosis was made according to the DSM-IV criteria [20] on the basis of the M.I.N.I interview, additional unstructured interviews, and information from medical records.

### LC-MS analysis of human plasma metabolites

LC-MS analysis was conducted based on our previous method [23]. Briefly, we extracted water-soluble metabolite from human plasma samples employing a modified Bligh and Dyer procedure. The plasma metabolites were analyzed by LC-MS based on both reverse phase ion-pair chromatography and hydrophilic interaction chromatography (HILIC) modes coupled with a triple quadrupole mass spectrometer LCMS-8040 (Shimadzu, Japan). For monitoring metabolites including intermediates in central metabolism and nucleotides, a reverse phase ion-pair chromatography was performed using an ACQUITY UPLC BEH C18 column (100 Å~ 2.1 mm, 1.7 μm particle size, Waters). The mobile phase consisted of solvent A (15 mM acetic acid and 10 mM tributylamine) and solvent B (methanol), and the column oven temperature was 40°C. The gradient elution program was as follows: a flow rate of 0.3 mL/min: 0–3 min, 0%B; 3–5 min, 0–40%B; 5–7 min, 40–100% B; 7–10 min, 100%B; 10.1–14 min, 0%B. Parameters for negative ESI mode under multiple reaction monitoring (MRM) were as follows; drying gas flow rate, 15 L/min; nebulizer gas flow rate, 3 L/min; DL temperature, 250°C; and heat block temperature, 400°C; collision energy (CE), 230kPa. On the other hand, for monitoring metabolites including amino acids, HILIC chromatography was performed using a Luna 3u HILIC 200A column (150 Å~ 2 mm, 3 μm particle size, Phenomenex). The mobile phase consisted of solvent A (10mM ammonium formate in water) and solvent B (9:1 of acetonitrile:10 mM ammonium formate in water), and the column oven temperature was 40°C. The gradient elution program was as follows: a flow rate of 0.3 mL/min: 0–2.5 min, 100%B; 2.5–4 min, 100–50%B; 4–7.5 min, 50–5% B; 7.5–10 min, 5%B; 10.1–12.5 min, 100%B. Parameters for positive and negative ESI mode under MRM were as described above.

### Metabolite data processing and statistical analysis

Metabolome data processing, including peak detection and retention time alignment, was carried out using LabSolutions LC-MS software program (Shimadzu, Japan). Totally, 123 metabolites were successfully detected in our targeted metabolome analysis. The multivariate data in each cohort study were pretreated with pareto scaling, and a partial-least-squares (PLS) regression model was created to isolate the SOD-associated biomarkers using the SIMCA 14.0 software (Umetrics, Sweden). On the other hand, machine learning modeling and statistical graphics were generated using R packages, including the ggplot2, e1071, randomForest, and ROC.

## Results

**Fig 2:**
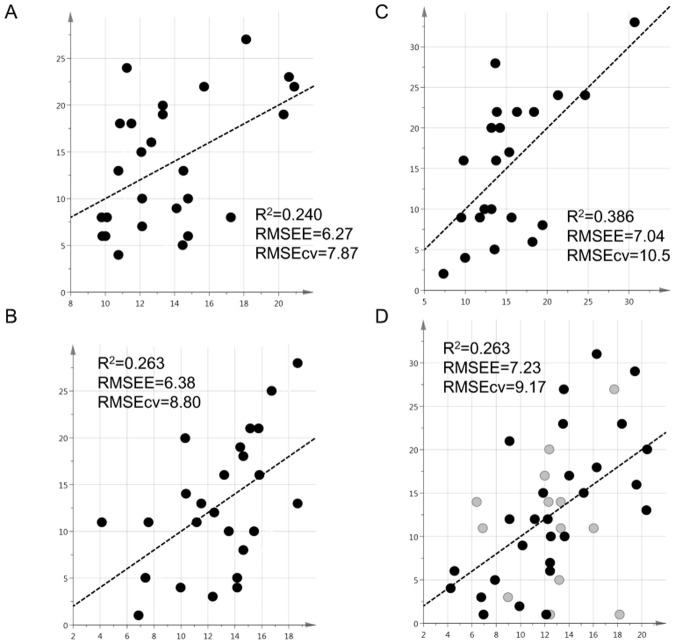
Plasma metabolome-based PLS regression model for PHQ-9 and HAMD-17 prediction. (A) For the medication-free plasma sample set of 26 patients, we conducted LC-MS-based metabolome analysis of water-soluble metabolites in plasma. Based on the 123 kinds of metabolites, the multivariate data sets were centered, scaled to Pareto and subjected to a multivariate statistical analysis using the SIMCA P+ ver. 14.0 software program (Umetrics, Sweden). The partial-least-squares (PLS) PHQ-9-regression model was created to identify the metabolites that associated with the severity of depression (SOD). The x-axis indicates the observed score of PHQ-9, while the y-axis indicates the predicted value of PHQ-9. (B) The HAMD-17-regression model was created based on the same data set 1. The x-axis indicates the observed score of HAMD-17, while the y-axis indicates the predicted value of HAMD-17. (C) Plasma samples from 23 medicated MDD patients (Data set 2) were subjected to LC-MS metabolome analysis and a HAMD-17-regression model was created as in (B). (D) Plasma samples of 41 patients from the third data set including both the medicated and non-medicated plasma samples, which were diagnosed with MDD (n = 27, black) and with bipolar disorder (n = 14, gray), were subjected to LC-MS metabolome/HAMD-17 regression analysis. R^2^, the square of Spearman’s correlation coefficient; RMSEE, root mean square error of estimation; RMSEcv, root mean square error of cross validation.

For the first sample set of a total of 26 medication-free psychiatric patients (Data set 1; average age (SD) = 30.7 (7.76)), we conducted LC-MS-based metabolome analysis of water-soluble metabolites sin plasma, and 123 metabolites were successfully detected. In order to explore the metabolites involved in the SOD, we created a PLS regression model to predict values for the total scores of either PHQ-9 or HAMD-17 (Fig 2A and 2B). We found that each model showed a fairly good correlation with either value R2 = 0.24 (PHQ-9) and R2 = 0.263 (HAMD-17) and the influential metabolites were listed almost as common (Table 1). This result has suggested that plasma metabolome analysis is a useful tool to evaluate SOD. The above metabolites, identified from medication-free samples, may however vary depending on drug administration. In order to address this possibility, we collected plasma samples from 23 MDD medicated patients (Data set 2; average age (SD) = 54.8 (13.5)) and conducted LC-MS metabolome analysis to create a HAMD-17-regression model with the same methodology. Interestingly, compared to the first data set, this second model showed stronger correlation R2 = 0.386 with HAMD-17 (Fig 2C), and 74% of influential metabolites were overlapped with the medication-free patient group (Table 1). These results indicated the possibility that quantitative validation of multiple metabolites reflect the severity of mood disorders irrespective of medication. The results strongly suggested that refining metabolite monitoring with fewer indicators could create an objective evaluation criteria as practical method. To validate this utility, we also obtained plasma samples of 41 patients from the third data set with medicated and un-medicated, all of which were diagnosed with MDD (n = 27; average age (SD) = 45.7 (11.0)) or with BD (n = 14; average age (SD) = 43.3 (12.0)) and performed a LC-MS metabolome/HAMD-17 regression analysis. In the third model, the correlation degree (Fig 2D, R2 = 0.263) and influential metabolites were almost the same as the first and second data sets (Table 1). Interestingly, we have revealed that five metabolites (3-hydroxybutyrate, betaine, citrate, creatinine, and gamma-aminobutyrate (GABA)), are commonly associated with SOD in all three independent data sets regardless of the presence or absence of medication and diagnostic difference. 3-hydroxybutyrate, the most contributing metabolite, is positively correlated with total score of HAMD-17 in all three independent data sets [Data set 1(R = 0.35), Data set 2 (R = 0.24), Data set 3 (R = 0.18)].

**Table 1:** Plasma metabolites primarily contributed to the respective PLS-regression model. The variable importance in projection (VIP) denotes the degree of contribution to the PLS regression model, whose scores greater than 1.0 can be considered important in given model. Metabolites displayed in bold style represent to commonly contribute to the three independent PLS regression models, thereby suggesting strongly associated with the underlying pathophysiology of depression.

	Data set-1 (PHQ-9)		Data set-1 (HAMD-17)		Data set-2 (HAMD-17)		Data set-3 (HAMD-17)	
	Drug free (N = 26)		Drug free (N = 25)		Medicated (N = 23)		Mix (N = 41)	
Rank	Metabolite	VIP	Metabolite	VIP	Metabolite	VIP	Metabolite	VIP
1	3HB*	6.11	3HB*	6.68	3HB*	6.58	3HB*	4.34
2	**Betaine**	4.43	Creatine	3.60	**Betaine**	4.12	Isoleucine	4.34
3	Proline	3.52	**Citrate**	3.49	Carnitine	3.21	**Betaine**	3.52
4	**Citrate**	3.37	**Betaine**	3.17	Acetylcarnitine	2.92	**Creatinine**	3.46
5	**Creatinine**	2.52	Lysine	2.44	**Creatinine**	2.55	Phenylalanine	3.35
6	Acetylcarnitine	2.45	Proline	2.18	Creatine	2.48	Acetylcarnitine	2.25
7	Isoleucine	1.66	Glutamine	2.06	Ornithine	2.35	**Citrate**	2.00
8	Phenylalanine	1.50	**Creatinine**	1.75	**Citrate**	1.50	GABA*	1.92
9	Glutamine	1.48	Carnitine	1.68	GABA*	1.47	Dimethylglycine	1.51
10	Lysine	1.29	Phenylalanine	1.57	Isoleucine	1.43	Proline	1.27
11	Carnitine	1.19	Taurine	1.36	Arginine	1.31	Lysine	1.19
12	Creatine	1.17	TMAO*	1.15	Norvaline	1.29	Argininosuccinate	1.07
13	GABA*	1.08	GABA*	1.03	TMAO*	1.11	Kynurenine	1.04

*3HB, 3-Hydroxybutyrate; GABA, gamma-aminobutyric acid; TMAO, trimethylamine N-oxide

Metabolites displayed in bold commonly contribute to the three independent PLS regression models.

**Fig 3:**
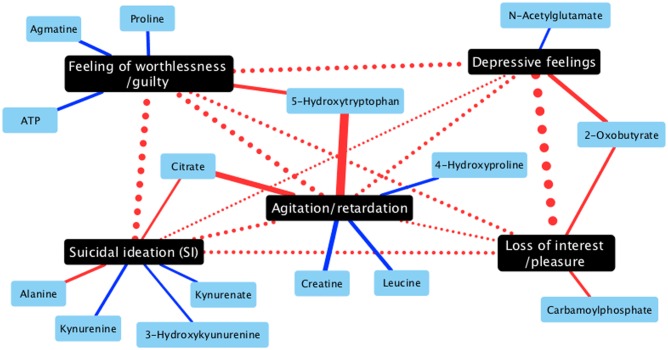
Correlation networks of HAMD-17 sub-scale and metabolites in medication-free cohort samples (Data set 1). Red solid/dot lines, positive correlation; blue solid/dot lines, negative correlation; Black nodes, HAMD-17 subscales; blue nodes, metabolites. Edge-width scales as the correlation coefficients in absolute value. The bolder the lines, the stronger the correlation between connected nodes is.

Next, to clarify what metabolites are associated with sub-symptoms of depression, we conducted a correlation analysis between sub-scores of PHQ-9 / HAMD-17 and 123 metabolites in medication-free samples (data set 1). Table 2 shows highly-associated metabolites in each sub-symptoms of depression. We have revealed that some different metabolites are differently associated with sub-symptoms of depression. Fig 3 shows correlation networks of HAMD-17 sub-scale and metabolites.

**Table 2:** Correlation between sub-scales of PHQ-9/HAMD-17 and plasma metabolites in medication-free cohort study (Data set-1). Metabolites which have moderate correlation (absolute correlation coefficient value >0.2) with reciprocally synonymous sub-scale of PHQ-9/HAMD-17 are listed. Gray shaded denotes negatively correlation with the respective sub-scales.

**Loss of interest/pleasure**				**Agitation/retardation**			
Metabolite	PHQ (1)	HAMD (2)		Metabolite	PHQ (8)	HAMD (16)	HAMD (17)
2-Oxobutyrate	0.368	0.283		5-Hydroxytryptophan	0.337	0.626	0.629
Acetylcarnitine	0.293	0.204		4-Hydroxyproline	-0.382	-0.290	-0.250
Proline	-0.365	-0.155		Creatine	-0.338	-0.390	-0.281
Carbamoylphosphate	0.385	0.280		Citrate	0.187	0.432	0.388
3-Methylhistidine	0.224	0.224		Leucine	-0.292	-0.336	-0.250
Urocanate	0.203	0.249		Fumarate	0.338	0.229	0.246
**Depressive feelings**				**Suicidal ideation (SI)**			
Metabolite	PHQ (2)	HAMD (1)		Metabolite	PHQ (9)	HAMD (11)	
N-Acetylglutamate	-0.207	-0.259		Kynurenine	-0.289	-0.284	
2-Oxobutyrate	0.383	0.361		Xanthurenate	-0.325	-0.201	
				3-Hydroxykynurenine	-0.226	-0.256	
**Feeling of worthlessness/guilty**				Kynurenate	-0.217	-0.256	
Metabolite	PHQ (6)	HAMD (10)		Xanthosine	-0.244	-0.250	
Proline	-0.082	-0.301		Citrate	0.224	0.252	
5-Hydroxytryptophan	0.196	0.324		Alanine	0.229	0.290	
Phosphoenolpyruvate	0.323	0.152					
ATP	-0.133	-0.299					
Agmatine	-0.146	-0.305					

For example, 2-oxobutyrate, one of the hydroxycarboxylic acids including 3-hydroxybutylate, are associated with both “loss of interest” and “depressive feelings”, while N-acelylglutamate is only associated with “depressive feelings”. Proline, 5-hydroxyltryptophan, phosphorenolpyruvate, ATP and agmatine are associated with “feeling of worthlessness/guilty”. 5-hydroxyltryptophan is also strongly associated with agitation/retardation. Interestingly, some kynurenine-pathway metabolites are negatively associated with “suicidal ideation (SI)”, and citrate and alanine are positively associated with SI. To validate the findings in data set 1 (medication-free data set), we also conducted correlation analysis using data set 2 & 3. As shown in Table 3, we have confirmed that citrate is fairly positively associated with SI and kynurenine-pathway metabolites (especially kynurenine and 3-hydroxykynurenine) are negatively associated with SI.

**Table 3:** Metabolites commonly associated with HAMD_SI. In three distinct cohort studies, four metabolites moderately and commonly correlate with HAMD_SI. Gray shaded denotes negatively correlation with the respective sub-scales.

Metabolite	Data set-1	Data set-2	Data set-3
Citrate	0.29	0.33	0.14
Kynurenine	-0.28	-0.30	-0.32
3-Hydroxykynurenine	-0.26	-0.25	-0.10
Kynurenate	-0.26	-0.27	0.08

**Fig 4:**
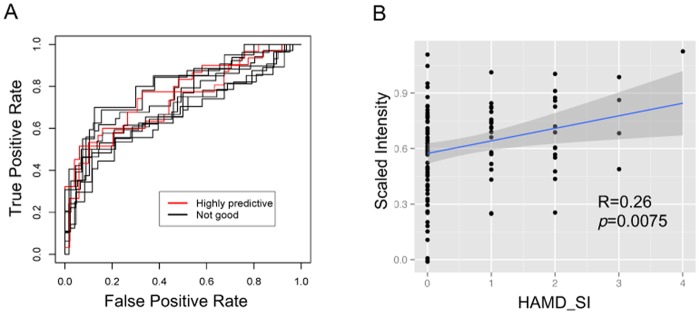
Classification and regression models for predicting suicidality and a grade of suicidal ideation in depressive patients. (**A**) Receiver-operating curve based on ten kinds of logistic regression models for classifying suicidality in depressive patients. Based on mixed data containing three independent data sets (Data set-1, Data set-2, and Data set-3), 10 kinds of distinct training data are created and subjected to building classification models discriminating between the depressive patients who have a sub-scale of suicidal ideation (HAMD_SI: HAMD-17_11> = 1) and not (HAMD_SI = 0) (details are described in S1 Table). Curves in red denote highly predictive models for test data set (true rate >0.7). (**B**) Significant correlation (R = 0.22, p = 0.028) between scored HAMD_SI and predictive scores of multiple linear regression model, based on standardized intensities of plasma citrate and kynurenine. A fitted linear regression line was depicted with 95% confidence area (gray shaded). Parameters of the model were described in S2 Table.

Differentiation between depression with SI or without SI is crucial in psychiatric and also primary care settings for suicide prevention. Thus, we have conducted a further analysis focusing on SI, and we have successfully created a classification model to discriminate depressive patients with or without SI (Fig 4A, S1 Table). Suicidality-classification models are created using conventional supervised machine learning approach. Among a total of 104 data, ten kinds of training data are used for creating predictive models by either logistic regression, support vector machine, or random forest procedure. Fitting ability was visualized by ROC curve and evaluated by values of area under the curve (AUC). Predictive abilities are evaluated by true rate of fitted test data set. Three models (two logistic regression and one support vector machine) denote highly predictive (AUC >0.7 and true rate>0.7). Furthermore, we have developed a pilot algorithm to predict a grade of SI using just two metabolites; citrate and kynurenine (R = 0.22, p = 0.028) (Fig 4B, S2 Table).

## Discussion

This is the first report to show that several plasma metabolites are commonly associated with SOD across three different sample sets regardless of the presence or absence of medication and diagnostic difference. In addition, we have successfully created a pilot classification model to predict suicidality, and an algorithm to estimate a grade of SI using only a few metabolites. The present results offer a potential utility for measuring metabolites as a novel objective tool for assessing SOD in clinical practice. Strong efforts to develop objective evaluation systems of depression have been conducted mainly using brain imaging/physiological techniques such as magnetic resonance imaging (MRI) and electroencephalogram (EEG). Recently, we have reported that female patients with schizophrenia and MDD can be discriminated by brain MRI [24]. Such brain-targeting assessment tools have been thought to be an appropriate method to grasp psychiatric pathophysiology, however these tools lack versatility. On the other hand, the present multi-center study to evaluate SOD by peripheral blood may have broad utility from psychiatric clinical practice to primary care settings.

The following metabolites may have strongly been associated with the underlying novel biological pathophysiology of SOD.

### 3-Hydroxybutyrate

The most highly influential metabolite 3-hydroxybutyrate (beta-hydroxybutyric acid) is one of the ketone bodies, and known as a marker of favoring lipid over glucose metabolism. 3-hydroxybutyrate is an energy source in the brain, and recent studies have suggested that this metabolite is associated with brain inflammation, especially deeply contributing to epilepsy [25, 26]. The present data has proposed that 3-hydroxybutyrate itself may control emotional systems in the brain via energy metabolite processes. Youm et al. reported that 3-hydroxybutyrate suppresses activation of the NLRP3 inflammasome in response to urate crystals, ATP and lipotoxic fatty acids, and revealed that 3-hydroxybutyrate reduces NLRP3 inflammasome-mediated interleukin (IL)-1β and IL-18 production in human monocytes [26]. The hydroxy-carboxylic acid receptor 2 (HCA2), one of the 3-hydroxybutyrate receptor, is known to be highly expressed in macrophage and also microglia in the brain [27], showing anti-inflammatory and neuroprotective actions [28, 29]. Interestingly, microglial abnormalities and dysfunction have been suggested under the pathophysiology of depression [14, 18, 30, 31]. The present finding has proposed that digging up the crosstalk between 3-hydroxybutyrate, microglia and inflammation will shed new light in order to further elucidate the molecular mechanism of depression.

### Betaine

Betaine (trimethylglycine) serves as osmolytes via the BHMT (Betaine-Homocysteine Methyltransferase) reaction, which converts homocysteine to methionine. Betaine is utilized for the treatment of homocystinuria. Upregulation of plasma homocysteine is associated with various physical illnesses such as arterial sclerosis, thus the eliminative role of betaine is suggested to be crucial to homeostasis [32, 33]. Increasing levels of homocysteine also induce inflammation in the brain via increasing oxidative stress and inflammatory cytokines, and a recent study revealed a lower plasma concentration of betaine in patients with first-episode schizophrenia [34]. Based on the above evidence, betaine is suggested to play important roles in expressing mental dysfunctions including depressive mood by regulating the BHMT reaction and the following systematic changes.

### Citrate

Citrate is one of the major multifunctional acids. In blood, citrate plays an important role in modulating the acid-base balance. Concentration of blood citrate is known to be associated with that of various blood hormones such as ACTH, insulin, adrenaline, noradrenaline, and epinephrine. These hormones are strongly linked to emotions and also psychiatric disorders especially depression [35–38], which support the significance of the present result. Citrate may be a key mood modulator by shifting the concentrations of mood-related hormones in blood. On the other hand, ketamine is now suggested to be a novel treatment for depression, and a mice metabolic profiling of ketamine injection has revealed a significant shift of citrate cycle in the hippocampus [39]. In addition, the blood coagulation system has been highlighted in the pathogenesis of depression [40–42]. In the present study, SOD (in particular, suicidal ideation) and blood citrate showed a positive correlation, suggesting that the chelating action by citrate may result in lowering blood coagulation ability to form divalent calcium ions and be a candidate pathway of suicide. Interestingly, a latest clinical study has revealed that cerebrospinal fluid metabolomics identifies a key role of isocitrate dehydrogenase in bipolar disorder, which has proposed the importance of the citric acid cycle in mood modification [43]. Further investigations are needed to clarify the roles of citrate peripherally and in the brain.

### Creatinine

Creatinine is a metabolite of creatine phosphate and an important energy store found in skeletal muscle. Creatinine is removed from the body entirely by the kidneys. Thus, creatinine has been widely utilized to measure renal function as a common blood test. Long term treatment of lithium chloride, which is widely used for the treatment of mood disorders especially mania, is known to cause higher blood creatinine as a result of kidney dysfunction. Epidemiological studies have shown the higher risks of depression and suicide in patients with chronic renal failure requiring hemodialysis [44, 45]. So far, a direct biological relationship among creatinine, renal dysfunction and psychiatric symptoms has not been understood, while the present data suggest a direct link among them.

### GABA

GABA (gamma-aminobutyric acid) is an amino acid, and has long been associated with wider mental illnesses from anxiety, depression to schizophrenia [46]. Lower levels of GABA in cerebrospinal fluid (CSF) was reported in patients with depressive disorders [47]. Recently, however, to our knowledge, no evidence has indicated the interaction of SOD and GABA. A recent study has shown that severity of psychic anxiety is correlated with lower CSF free GABA but not related to SOD [48]. The present result is the first data to show the interaction of SOD and GABA.

We have also revealed some other metabolites are associated with sub-symptoms of depression. Agmatine is known to have antidepressant-like effects in rodent studies [49, 50] and is proposed to be a therapeutic target for stress-related disorders such as depression, anxiety and post-traumatic stress disorder [51, 52]. Herein, we have shown that agmatine is negatively correlated with guilt, proposing that agmatine may be a therapeutic target for guilt-proneness.

Decades of evidence suggests that the brain neurotransmitter serotonin is incriminated in the brain of patients with depression [53, 54], and the relationship between tryptophan-metabolism intermediates and depression has recently been highlighted [55, 56]. Tryptophan metabolism is divided into serotonin and kynurenine pathways. In this study, the serotonin biosynthesis intermediate 5-hydroxytryptophan is strongly correlated with agitation/retardation, which may reflect some kind of abnormality in the serotonin pathway during agitation/retardation. On the other hand, we have shown that reduction in the kynurenine pathway intermediates (kynurenine, kynurenate, and 3-hydroxykynurenine) is involved in SI. The kynurenine pathway is strongly liked to modulation of glial cells in the brain, and abnormalities of the kynurenine pathway and glial maladaptive activation have recently been highlighted to understand the underlying pathophysiology of various psychiatric conditions including depression [14, 30, 57]. Some plasma metabolites related to the kynurenine pathway are downregulated during higher SI in the present study. Previously several reports have indicated the upregulation of kynurenine metabolite pathway in suicidality whilst other reports have shown contradictory findings [58–60]. Further clinical investigations, especially focusing on time-course analysis should be conducted.

In addition to the above-discussed metabolites, we presented many other metabolites in the present study (Tables 1 and 2), which may also contribute to the underlying pathophysiology of SOD and sub-symptoms of depression. Just recently, other researchers have also reported utilities of plasma metabolomics focusing on depression. Liu X. et al. have reported metabolomic analysis using plasma samples from patients with MDD and healthy controls, showing that levels of acyl carnitines, ether lipids, and tryptophan pronouncedly decreased, whereas lysophosphatidylcholines, lysophosphatidylethanolamines, and phosphatidylethanolamines markedly increased in MDD subjects as compared with the healthy controls [61]. On the other hand, Liu Y. et al. have reported metabolomic analysis focusing on two clinical subtypes within MDD, “melancholic depression” and “anxious depression”, and revealed that melancholic depression is associated with changes in amino acids, catecholamines, lipids, stress hormones, and immune-related metabolites, and they have also shown that metabolite data can significantly differentiate melancholic depressive patients from healthy controls [62].

### Limitation and future perspectives

In the present study, SI was measured only by PHQ-9 and/or HAMD-17, which may influence our outcomes. To establish a more objective measurement of suicidal risk, the analysis of the metabolite profile of patients who experience suicide attempts is warranted. In this pilot investigation, we could not measure/analyze metabolites in healthy persons (because we did not collect PHQ-9/HAMD-17 data in non-clinical subjects), thus no control group exists, which is a limitation in this study. In addition, the present clinical data lack the age of onset and the duration of illness, which may influence the present data. The present pilot findings should be validated by wider/larger population studies including non-clinical subjects (control groups). Case-control studies are also needed. This study was conducted among Japanese samples, and international validation should be conducted. We should explore the biological impact of these metabolites on depressive symptoms by utilizing a cross species study model with human and rodents. Another limitation is that we did not check as to whether the present blood samples were collected under fasting or non-fasting conditions. Levels of some plasma metabolites are easily affected by diet. We expect that therapeutic interventions to modify the present finding metabolites by diet and/or medications may be a novel therapeutic target for depression, and further longitudinal studies are warranted [63]. Despite these limitations, the present study has shed new light on blood biomarker screening of SOD by plasma metabolites. Evaluating severity of depression (SOD), especially suicidal ideation (SI), is crucial not only in psychiatric settings but also primary care and social situations to prevent suicidal incidents [64, 65], and hopefully SOD and SI evaluation systems with plasma metabolites will be developed for use in simple and easy evaluation kits for clinical practices and at medical checkups in the future.

## Supporting Information

S1 Table Machine learning models for discriminating depressive patients with suicidality. Suicidality-classification models are created using conventional supervised machine learning approach. Among a total of 104 data, ten kinds of training data are used for creating predictive models by either logistic regression, support vector machine, or random forest procedure. Fitting ability was visualized by ROC curve (Fig 4A) and evaluated by values of area under the curve (AUC). Predictive abilities are evaluated by true rate of fitted test data set. Gray shaded models denote highly predictive (AUC >0.7 and true rate>0.7). All random forest models seem to be as over-fitted (AUC = 1) and thereby result in poor performance.
(DOCX)

S2 Table Parameters for stepwise multiple linear regression model for predicting a grade of suicide ideation in depressive patients. Among the HAMD_SI-correlated metabolites, stepwise regression was carried out and citrate and kynurenine are selected as variables essential for suicidal prediction. In the model, intercept and kynurenine are shown as significant (*: *p* <0.05).
(DOCX)
