# Supplementary material for: Plasma Metabolites Predict Severity of Depression and Suicidal Ideation in Psychiatric Patients-A Multicenter Pilot Analysis
Source: PLoS One. 2016 Dec 16;11(12):e0165267. doi: 10.1371/journal.pone.0165267 (PMC5161310; doi:10.1371/journal.pone.0165267)
Supplement: S1 Table — Suicidality-classification models are created using conventional supervised machine learning approach. Among a total of 104 data, ten kinds of training data are used for creating predictive models by either logistic regression, support vector machine, or random forest procedure. Fitting ability was visualized by ROC curve (Fig 4A) and evaluated by values of area under the curve (AUC). Predictive abilities are evaluated by true rate of fitted test data set. Gray shaded models denote highly predictive (AUC >0.7 and true rate>0.7). All random forest models seem to be as over-fitted (AUC = 1) and thereby result in poor performance. (DOCX) [file pone.0165267.s001.docx]

**S1 Table. Machine learning models for discriminating depressive patients with suicidality**

Suicidality-classification models are created using conventional supervised machine learning approach. Among a total of 104 data, ten kinds of training data are used for creating predictive models by either logistic regression, support vector machine, or random forest procedure. Fitting ability was visualized by ROC curve (**Fig 4A**) and evaluated by values of area under the curve (AUC). Predictive abilities are evaluated by true rate of fitted test data set. Gray shaded models denote highly predictive (AUC >0.7 and true rate>0.7). All random forest models seem to be as over-fitted (AUC=1) and thereby result in poor performance.

| ID | Total Data | Training Model | | Model Fit to Test Data | | |
| --- | --- | --- | --- | --- | --- | --- |
|  |  | Number of data | AUC | Number of data | TRUE | True Rate (%) |
| Logistic regression_1 | 104 | 90 | **0.70** | 14 | 6 | 42.9 |
| Logistic regression_2 | 104 | 90 | **0.80** | 14 | 5 | 35.7 |
| Logistic regression_3 | 104 | 90 | **0.76** | 14 | 5 | 35.7 |
| Logistic regression_4 | 104 | 80 | **0.68** | 24 | 17 | **70.8** |
| Logistic regression_5 | 104 | 80 | **0.75** | 24 | 17 | **70.8** |
| Logistic regression_6 | 104 | 80 | **0.75** | 24 | 18 | **75.0** |
| Logistic regression_7 | 104 | 70 | **0.68** | 34 | 19 | 55.9 |
| Logistic regression_8 | 104 | 70 | **0.74** | 34 | 18 | 52.9 |
| Logistic regression_9 | 104 | 70 | **0.66** | 34 | 27 | 79.4 |
| Logistic regression_10 | 104 | 60 | **0.81** | 44 | 23 | 52.3 |
| Support vector machine_1 | 104 | 90 | 0.60 | 14 | 4 | 28.6 |
| Support vector machine_2 | 104 | 90 | 0.68 | 14 | 5 | 35.7 |
| Support vector machine_3 | 104 | 90 | **0.76** | 14 | 6 | 42.9 |
| Support vector machine_4 | 104 | 80 | **0.69** | 24 | 14 | 58.3 |
| Support vector machine_5 | 104 | 80 | **0.79** | 24 | 16 | 66.7 |
| Support vector machine_6 | 104 | 80 | **0.74** | 24 | 19 | **79.2** |
| Support vector machine_7 | 104 | 70 | 0.70 | 34 | 16 | 47.1 |
| Support vector machine_8 | 104 | 70 | 0.69 | 34 | 21 | 61.8 |
| Support vector machine_9 | 104 | 70 | **0.70** | 34 | 21 | 61.8 |
| Support vector machine_10 | 104 | 60 | **0.71** | 44 | 21 | 47.7 |
| Random forest_1 | 104 | 90 | 1.00 | 14 | 4 | 28.6 |
| Random forest_2 | 104 | 90 | 1.00 | 14 | 5 | 35.7 |
| Random forest_3 | 104 | 90 | 1.00 | 14 | 6 | 42.9 |
| Random forest_4 | 104 | 80 | 1.00 | 24 | 14 | 58.3 |
| Random forest_5 | 104 | 80 | 1.00 | 24 | 16 | 66.7 |
| Random forest_6 | 104 | 80 | 1.00 | 24 | 17 | 70.8 |
| Random forest_7 | 104 | 70 | 1.00 | 34 | 19 | 55.9 |
| Random forest_8 | 104 | 70 | 1.00 | 34 | 24 | 70.6 |
| Random forest_9 | 104 | 70 | 1.00 | 34 | 23 | 67.6 |
| Random forest_10 | 104 | 60 | 1.00 | 44 | 23 | 52.3 |
