# Supplementary material for: Plasma Metabolites Predict Severity of Depression and Suicidal Ideation in Psychiatric Patients-A Multicenter Pilot Analysis
Source: PLoS One. 2016 Dec 16;11(12):e0165267. doi: 10.1371/journal.pone.0165267 (PMC5161310; doi:10.1371/journal.pone.0165267)
Supplement: S2 Table — Among the HAMD_SI-correlated metabolites, stepwise regression was carried out and citrate and kynurenine are selected as variables essential for suicidal prediction. In the model, intercept and kynurenine are shown as significant (*: p <0.05). (DOCX) [file pone.0165267.s002.docx]

**S2 Table. Parameters for stepwise multiple linear regression model for predicting a grade of suicide ideation in depressive patients.**

Among the HAMD_SI-correlated metabolites, stepwise regression was carried out and citrate and kynurenine are selected as variables essential for suicidal prediction. In the model, intercept and kynurenine are shown as significant (*: *p* <0.05).

| Coefficients | Estimate | Std.Error | *t* value | *p* value |  |
| --- | --- | --- | --- | --- | --- |
| Intercept | 0.62 | 0.09 | 6.94 | 0.00 | * |
| Citrate | 0.13 | 0.09 | 1.42 | 0.16 |  |
| Kynurenine | -0.21 | 0.09 | -2.30 | 0.02 | * |
| R: 0.22 |  |  |  |  |  |
| p-value: 0.02879 | |  |  |  |  |
